# Supplementary material for: Optineurin modulates the maturation of dendritic cells to regulate autoimmunity through JAK2-STAT3 signaling
Source: Nat Commun. 2021 Oct 27;12:6198. doi: 10.1038/s41467-021-26477-4 (PMC8551263; doi:10.1038/s41467-021-26477-4)
Supplement: Supplementary file 1 — Supplementary Information [file 41467_2021_26477_MOESM1_ESM.pdf]

Supplementary Information

Optineurin modulates the maturation of dendritic cells to regulate autoimmunity through JAK2-STAT3 signaling

Jiajia Wang et al.

Corresponding authors: Qinjie Weng

Supplementary Figures

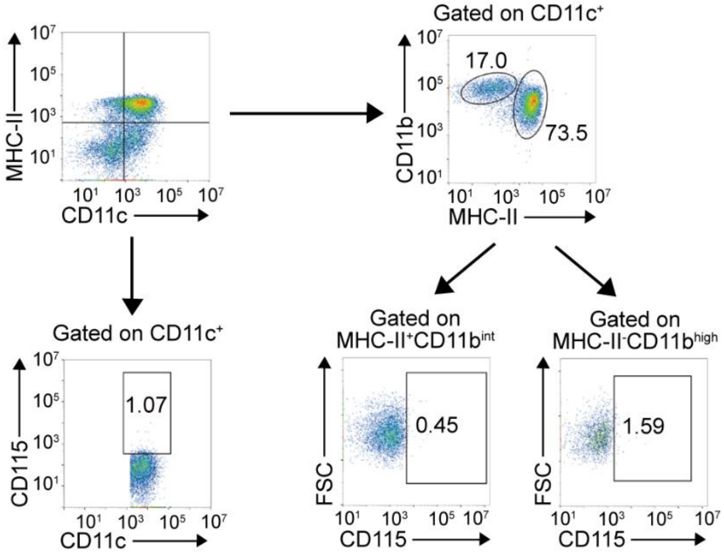

Supplementary Figure 1. Analysis of CD115 expression in BMDCs culture system.
Flow cytometry analysis of the frequency of CD115^{+} cells in CD11c^{+}, CD11c^{+}MHC-II^{+}CD11b^{int} or CD11c^{+}MHC-II^{-}CD11b^{high} cells. Data are representative of three independent experiments.

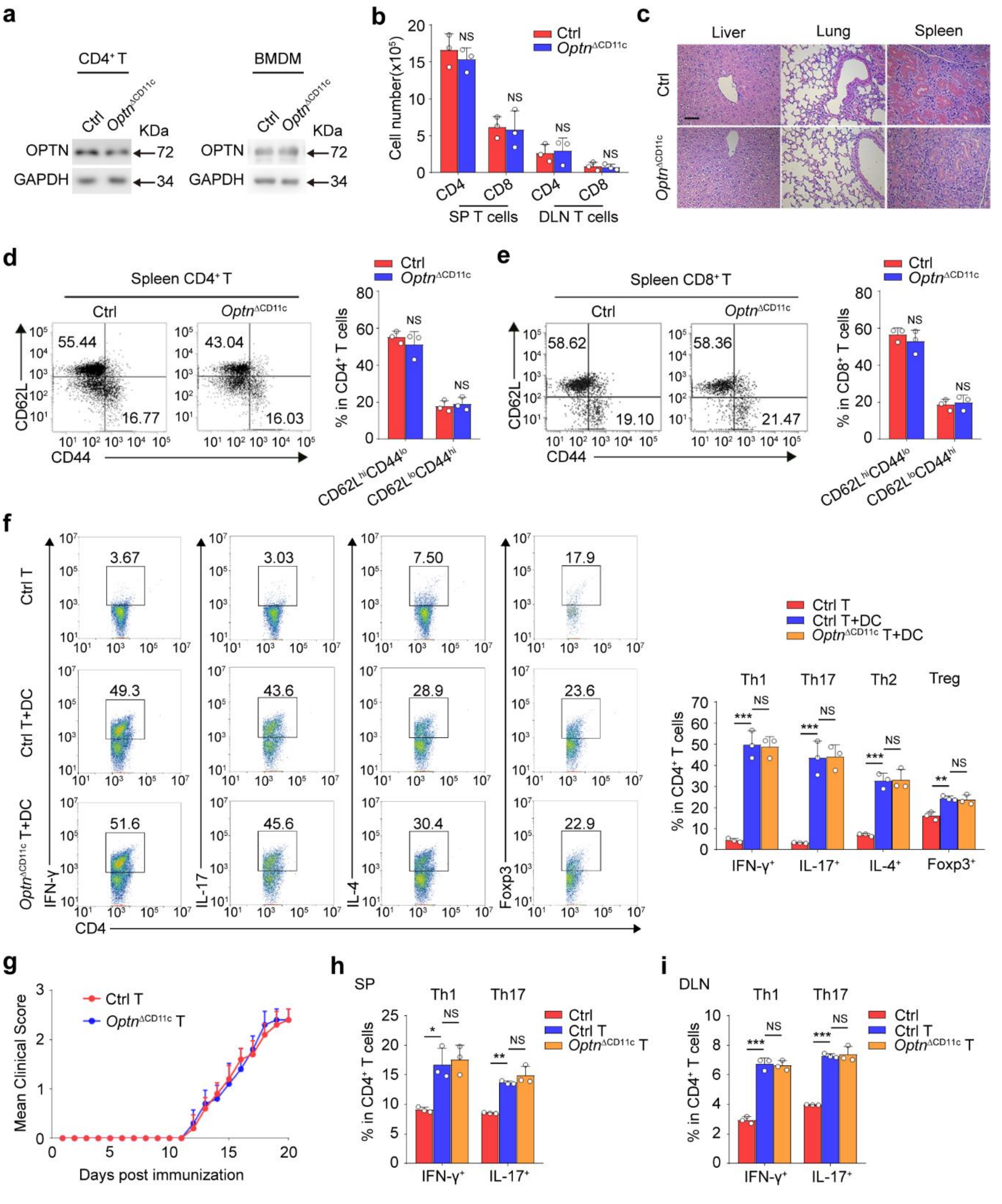

Supplementary Figure 2. ***Optn*$^{\Delta CD11c}$ mice have normal T cell function.**

a Western blotting analysis of $CD4^+$ T cells and BMDMs from Ctrl and *Optn*$^{\Delta CD11c}$ mice. $n = 3$ independent experiments. **b** Flow cytometry analysis for the number of $CD4^+$ and $CD8^+$ T cells in spleen (SP) and draining lymph nodes (DLN) from Ctrl and *Optn*$^{\Delta CD11c}$ mice. $n = 3$ independent animals, $P_{(SP\text{-}CD4)} = 0.4527$, $P_{(SP\text{-}CD8)} = 0.8328$, $P_{(DLN\text{-}CD4)} = 0.7984$, $P_{(DLN\text{-}CD8)} = 0.8122$. **c** HE staining of different tissues from Ctrl and *Optn*$^{\Delta CD11c}$ mice. Scale bar, 100 μm. $n = 3$ independent animals. **d, e** Flow cytometry analysis of the frequency of naive ($CD44^{lo}CD62L^{hi}$) and memory-

like ($CD44^{hi}CD62L^{lo}$) $CD4^+$ (**d**) and $CD8^+$ (**e**) T cells in spleen from Ctrl and *Optn*$^{\Delta CD11c}$ mice. $n = 3$ independent animals, $P_{(CD62L^{hi}CD44^{lo})} = 0.4138$, $P_{(CD62L^{lo}CD44^{hi})} = 0.6686$ in (**d**); $P_{(CD62L^{hi}CD44^{lo})} = 0.4025$, $P_{(CD62L^{lo}CD44^{hi})} = 0.6892$ in (**e**). **f** Flow cytometry analysis of the differentiation of T cells from Ctrl or *Optn*$^{\Delta CD11c}$ mice after co-culture with Ctrl DCs pulsed with $MOG_{35\text{-}55}$ (20 μg/mL) for 2 days. $n = 3$ independent experiments, $P_{(Ctrl)} = 5.6E\text{-}05$, $P_{(Optn^{\Delta CD11c}\ T+DC)} = 0.9637$ of IFN-γ; $P_{(Ctrl)} = 0.0003$, $P_{(Optn^{\Delta CD11c}\ T+DC)} = 0.9936$ of IL-17; $P_{(Ctrl)} = 0.0003$, $P_{(Optn^{\Delta CD11c}\ T+DC)} = 0.9895$ of IL-4; $P_{(Ctrl)} = 0.0035$, $P_{(Optn^{\Delta CD11c}\ T+DC)} = 0.8783$ of Foxp3. **g-i** C57BL/6 mice (8-week-old, female) were adoptive transferred with T cells that from Ctrl or *Optn*$^{\Delta CD11c}$ mice and stimulated with Ctrl DCs pulsed with $MOG_{35\text{-}55}$ for 2 days for employing EAE. Mean clinical scores are shown in (**g**). $n = 5$ independent animals. Flow cytometry analysis of T cells in spleen (**h**) and DLN (**i**). $n = 3$ independent animals, $P_{(Ctrl)} = 0.0112$, $P_{(Optn^{\Delta CD11c}\ T)} = 0.8756$ of IFN-γ in (**h**); $P_{(Ctrl)} = 0.0012$, $P_{(Optn^{\Delta CD11c}\ T)} = 0.2997$ of IL-17 in (**h**); $P_{(Ctrl)} = 1.9E\text{-}05$, $P_{(Optn^{\Delta CD11c}\ T)} = 0.9085$ of IFN-γ in (**i**); $P_{(Ctrl)} = 3.4E\text{-}05$, $P_{(Optn^{\Delta CD11c}\ T)} = 0.9364$ of IL-17 in (**i**). Data are presented as means ± SD. *, $P < 0.05$; **, $P < 0.01$; ***, $P < 0.001$; NS, not significant. Unpaired two-tailed Student's t-test for (**b, d, e**); one-way ANOVA Tukey's post-hoc analysis for (**f, h, i**). Source data are provided in Source Data file.

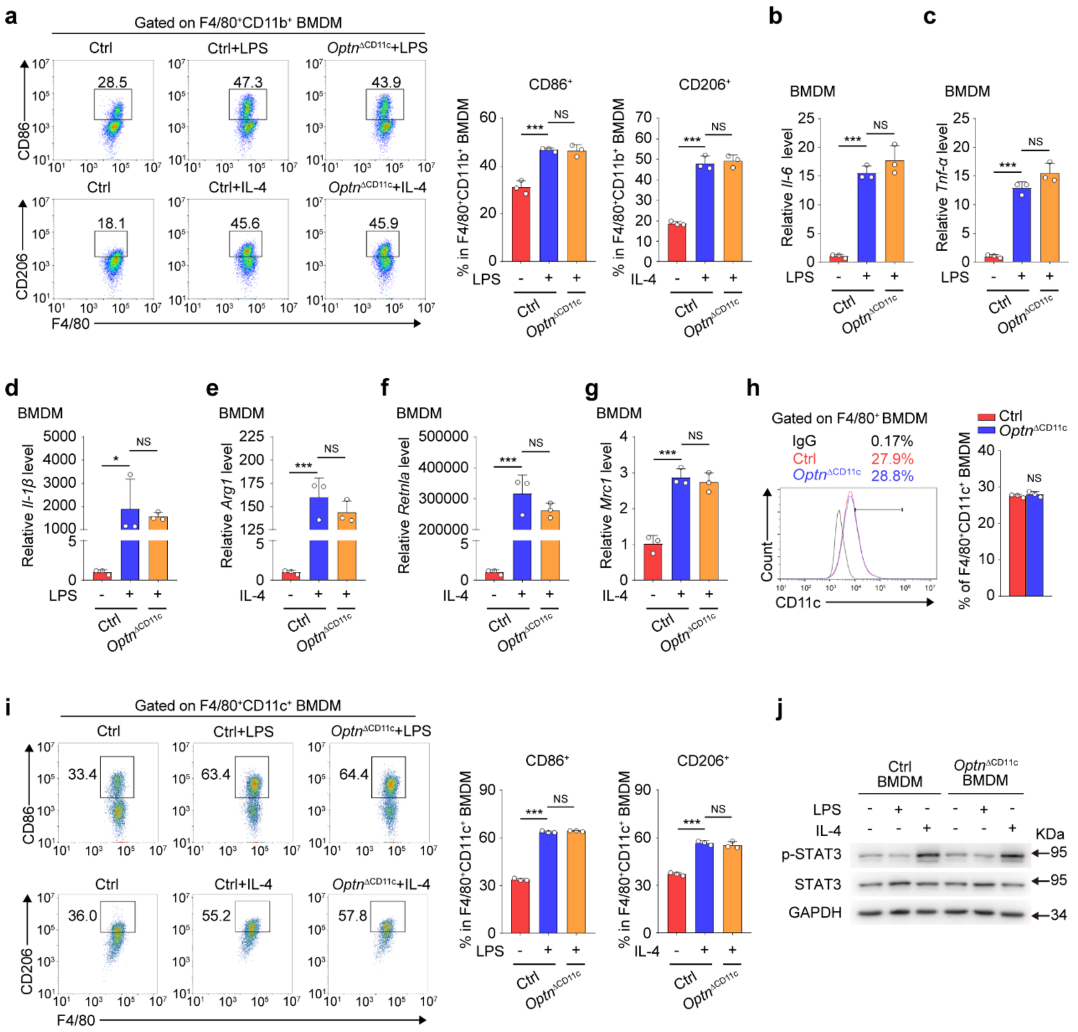

Supplementary Figure 3. ***Optn*$^{\Delta CD11c}$ mice have normal macrophage function.**

a Flow cytometry analysis of the frequency of M1 CD86^{+} BMDMs after LPS stimulus (50 ng/mL, 24 h) and M2 CD206^{+} BMDMs after IL-4 stimulus (20 ng/mL, 24 h) from Ctrl or *Optn*$^{\Delta CD11c}$ mice. $n = 3$ independent experiments, $P_{(Ctrl)} = 0.0003$, $P_{(Optn^{\Delta CD11c}+LPS)} = 0.9808$ of CD86; $P_{(Ctrl)} = 2.5E\text{-}05$, $P_{(Optn^{\Delta CD11c}+LPS)} = 0.8786$ of CD206. **b-g** mRNA levels of M1 macrophages markers (**b-d**) and M2 macrophages markers (**e-g**) in BMDMs from Ctrl or *Optn*$^{\Delta CD11c}$ mice stimulated with LPS or IL-4. $n = 3$ independent experiments, $P_{(Ctrl)} = 9.5E\text{-}05$, $P_{(Optn^{\Delta CD11c}+LPS)} = 0.3134$ in (**b**); $P_{(Ctrl)} = 4.5E\text{-}05$, $P_{(Optn^{\Delta CD11c}+LPS)} = 0.0806$ in (**c**); $P_{(Ctrl)} = 0.0485$, $P_{(Optn^{\Delta CD11c}+LPS)} = 0.8613$ in (**d**); $P_{(Ctrl)} = 2.2E\text{-}05$, $P_{(Optn^{\Delta CD11c}+LPS)} = 0.3872$ in (**e**); $P_{(Ctrl)} = 0.00011$, $P_{(Optn^{\Delta CD11c}+LPS)} = 0.2554$ in (**f**); $P_{(Ctrl)} = 0.0002$, $P_{(Optn^{\Delta CD11c}+LPS)} = 0.7783$ in (**g**). **h** Flow cytometry analysis of the frequency of CD11c^{+}F4/80^{+} cells in BMDMs from Ctrl or *Optn*$^{\Delta CD11c}$ mice. $n = 3$ independent experiments, $P = 0.4044$. **i** Flow cytometry analysis of the frequency of CD86^{+}CD11c^{+} and CD206^{+}CD11c^{+} cells in BMDMs from Ctrl or *Optn*$^{\Delta CD11c}$ mice stimulated with LPS or IL-4. $n = 3$ independent experiments, $P_{(Ctrl)} = 5.5E\text{-}11$, $P_{(Optn^{\Delta CD11c}+LPS)} = 0.5294$ of CD86; $P_{(Ctrl)} = 1.3E\text{-}05$, $P_{(Optn^{\Delta CD11c}+LPS)} = 0.5747$ of CD206. **j** Expression of (p-)STAT3 in whole-cell lysates of BMDMs from Ctrl or *Optn*$^{\Delta CD11c}$ mice stimulated with LPS or IL-4. $n = 3$ independent experiments. Data are presented as means ± SD. *, $P < 0.05$; ***, $P < 0.001$; NS, not significant. Unpaired two-tailed Student's t-test for (**h**); one-way ANOVA Tukey's post-hoc analysis for (**a-g, i**). Source data are provided in Source Data file.

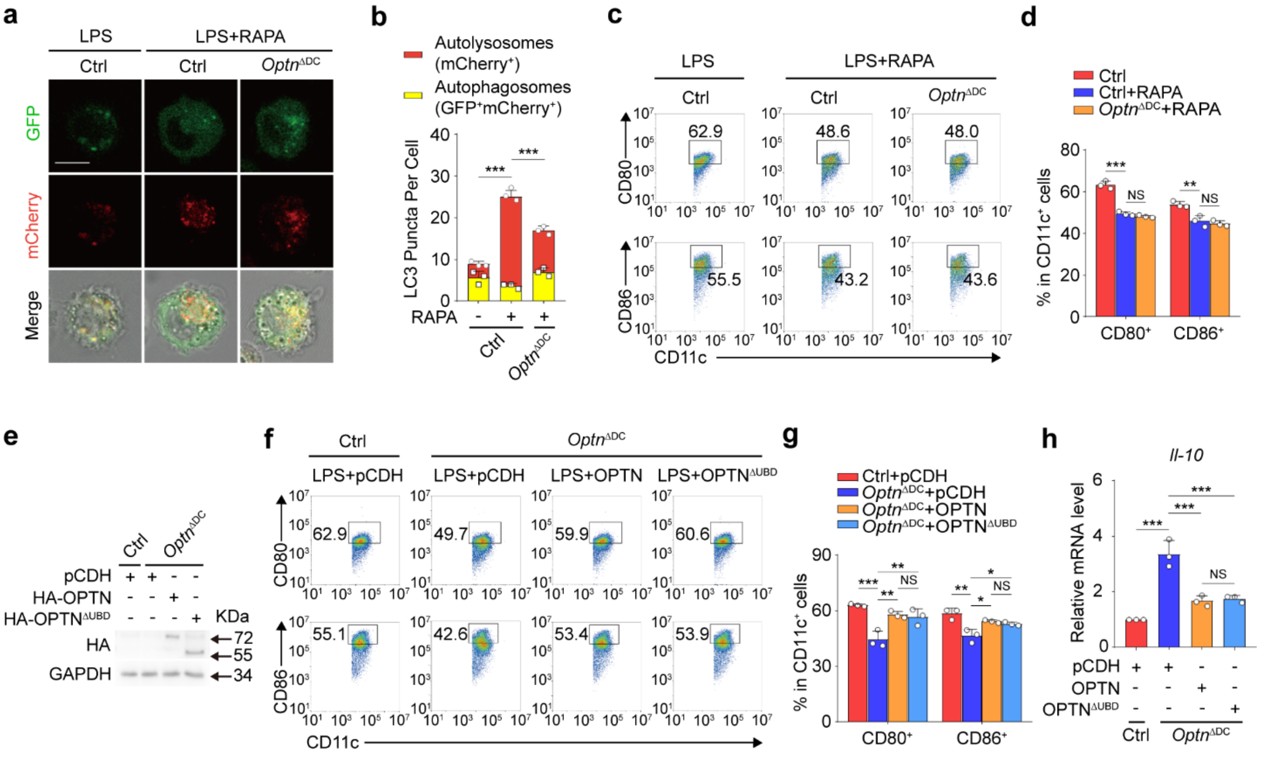

Supplementary Figure 4. OPTN mediated DC maturation is independent of its autophagy and ubiquitin functions.

a, b Immunofluorescence of GFP (green) and mCherry (red) in Ctrl and *Optn* deficient BMDCs stimulated with RAPA for 24 h after transfection with Lenti-GFP-mCherry-LC3B plasmid. Scale bars, 10 μm. $n = 3$ independent experiments, $P_{(\text{Ctrl})} = 2.4\text{E-}06$, $P_{(Optn^{\Delta DC}\text{+RAPA})} = 3.8\text{E-}05$ of autolysosomes. **c, d** Expression of CD80 and CD86 were analyzed by flow cytometry after stimulating with RAPA for 24 h in Ctrl or *Optn* deficient BMDCs. $n = 3$ independent experiments, $P_{(\text{Ctrl})} = 2.7\text{E-}05$, $P_{(Optn^{\Delta DC}\text{+RAPA})} = 0.5027$ of CD80; $P_{(\text{Ctrl})} = 0.0060$, $P_{(Optn^{\Delta DC}\text{+RAPA})} = 0.7278$ of CD86. **e** Western blotting to verify the overexpression of OPTN or OPTN$^{\Delta UBD}$ in *Optn* deficient BMDCs. $n = 3$ independent experiments. **f, g** Expression of CD80 and CD86 were analyzed by flow cytometry after transfection with OPTN or OPTN$^{\Delta UBD}$ plasmid in *Optn* deficient BMDCs. $n = 3$ independent experiments, $P_{(Optn^{\Delta DC}\text{+pCDH-Ctrl+pCDH})} = 0.0006$, $P_{(Optn^{\Delta DC}\text{+OPTN-}Optn^{\Delta DC}\text{+pCDH})} = 0.0052$, $P_{(Optn^{\Delta DC}\text{+OPTN}^{\Delta UBD}\text{-}Optn^{\Delta DC}\text{+pCDH})} = 0.0085$, $P_{(Optn^{\Delta DC}\text{+OPTN}^{\Delta UBD}\text{-}Optn^{\Delta DC}\text{+OPTN})} = 0.9764$ of CD80; $P_{(Optn^{\Delta DC}\text{+pCDH-Ctrl+pCDH})} = 0.0012$, $P_{(Optn^{\Delta DC}\text{+OPTN-}Optn^{\Delta DC}\text{+pCDH})} = 0.0164$, $P_{(Optn^{\Delta DC}\text{+OPTN}^{\Delta UBD}\text{-}Optn^{\Delta DC}\text{+pCDH})} = 0.0441$, $P_{(Optn^{\Delta DC}\text{+OPTN}^{\Delta UBD}\text{-}Optn^{\Delta DC}\text{+OPTN})} = 0.8864$ of CD86. **h** Expression of *Il-10* were analyzed by qRT-PCR after transfection with OPTN or OPTN$^{\Delta UBD}$ plasmid in *Optn* deficient BMDCs. $n = 3$ independent experiments, $P_{(Optn^{\Delta DC}\text{+pCDH-Ctrl+pCDH})} = 2.6\text{E-}05$, $P_{(Optn^{\Delta DC}\text{+OPTN-}Optn^{\Delta DC}\text{+pCDH})} = 0.0003$, $P_{(Optn^{\Delta DC}\text{+OPTN}^{\Delta UBD}\text{-}Optn^{\Delta DC}\text{+pCDH})} = 0.0004$, $P_{(Optn^{\Delta DC}\text{+OPTN}^{\Delta UBD}\text{-}Optn^{\Delta DC}\text{+OPTN})} = 0.9823$. Data are presented as means ± SD. *, $P < 0.05$; **, $P < 0.01$; ***, $P < 0.001$; NS, not significant. One-way ANOVA Tukey's post-hoc analysis. Source data are provided in Source Data file.

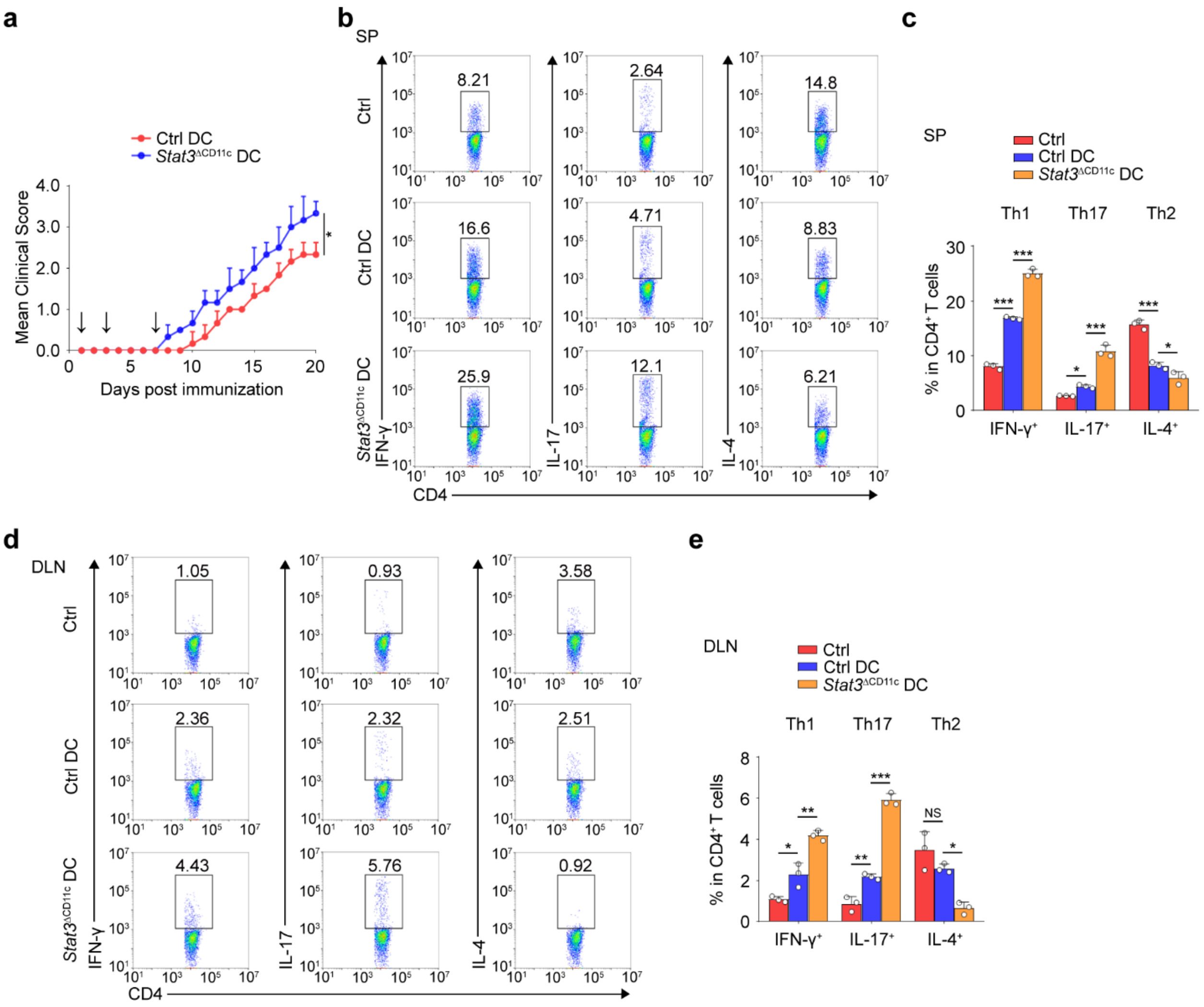

Supplementary Figure 5. Adoptive transfer of *Stat3* null BMDCs aggravates autoimmunity in EAE mice.

a Mean clinical scores of $MOG_{35–55}$-induced EAE mice (8-week-old, female) that adoptive transfer of Ctrl or *Stat3* knockout BMDCs. $n = 3$ independent animals, $P = 0.0132$. **b-e** Frequency of Th1, Th17 and Th2 cells in the spleen (SP) (**b, c**) and draining lymph nodes (DLN) (**d, e**) of ctrl or adoptive transfer EAE mice on dpi 20, shown as a representative plot (**b, d**) and summary graphs (**c, e**). $n = 3$ independent animals, $P_{(Ctrl)} = 3.0E\text{-}06$, $P_{(Stat3^{\Delta CD11c}\ DC)} = 4.7E\text{-}06$ of IFN-γ in (**c**); $P_{(Ctrl)} = 0.0477$, $P_{(Stat3^{\Delta CD11c}\ DC)} = 5.8E\text{-}05$ of IL-17 in (**c**); $P_{(Ctrl)} = 0.0001$, $P_{(Stat3^{\Delta CD11c}\ DC)} = 0.0489$ of IL-4 in (**c**); $P_{(Ctrl)} = 0.0169$, $P_{(Stat3^{\Delta CD11c}\ DC)} = 0.0018$ of IFN-γ in (**e**); $P_{(Ctrl)} = 0.0031$, $P_{(Stat3^{\Delta CD11c}\ DC)} = 8.5E\text{-}06$ of IL-17 in (**e**); $P_{(Ctrl)} = 0.2183$, $P_{(Stat3^{\Delta CD11c}\ DC)} = 0.0140$ of IL-4 in (**e**). Data are presented as means ± SD. *, $P < 0.05$; **, $P < 0.01$; ***, $P < 0.001$; NS, not significant. Unpaired two-tailed Student's t-test for (**a**); one-way ANOVA Tukey's post-hoc analysis for (**c, e**). Source data are provided in Source Data file.

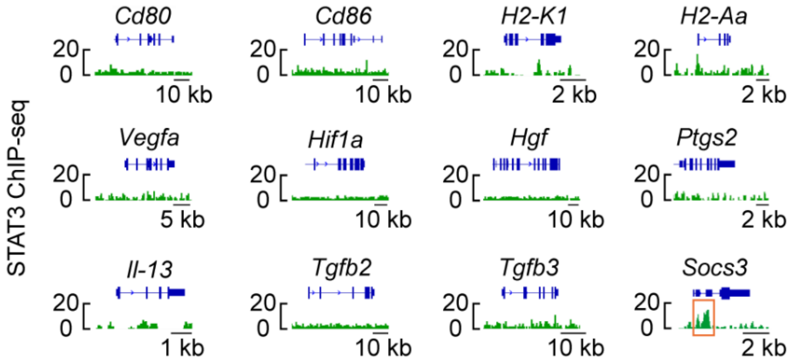

Supplementary Figure 6. ChIP-seq analysis of STAT3 target genes in DCs.
Peak tracking analysis of STAT3 ChIP-seq data on selected genes as shown by IGV software from GEO dataset (GSE27161).

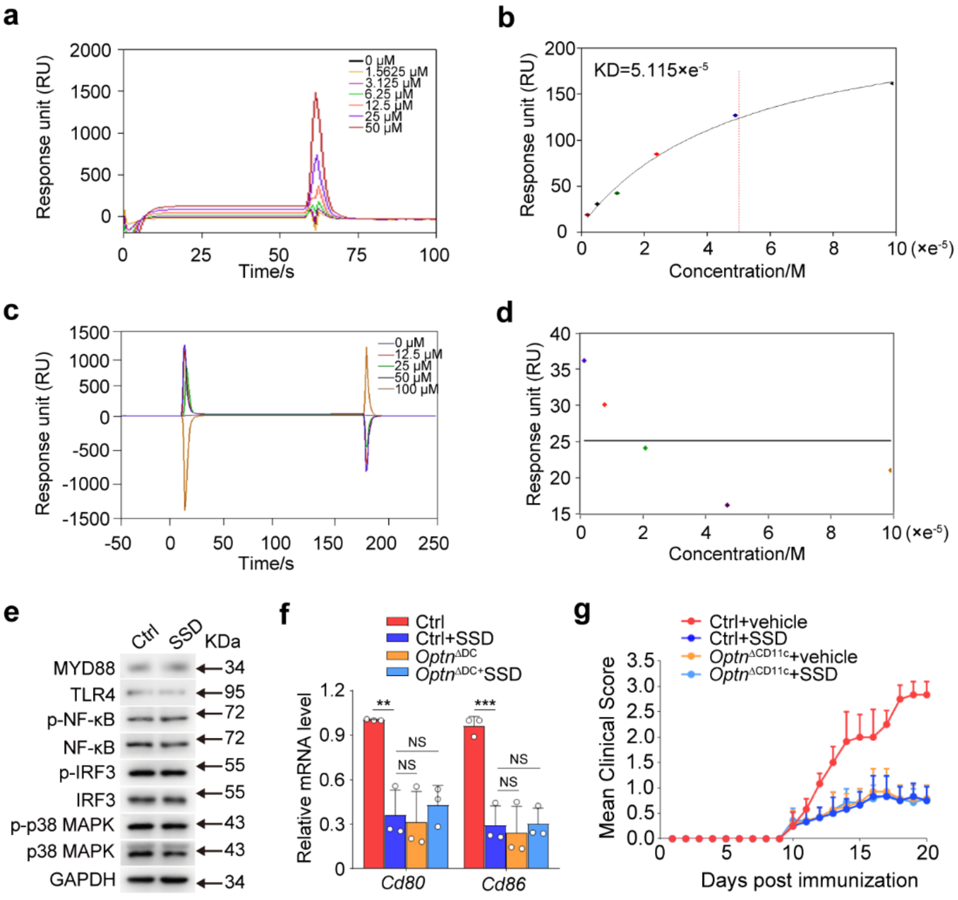

Supplementary Figure 7. SSD specifically regulates OPTN to relieve EAE.

a-d SPR assay characterization of the binding of SSD to OPTN (**a, b**) or UBA3 (**c, d**) using a BIAcore 3000 system. BIAcore diagram (**a, c**), and saturation curves (**b, d**) of SSD binding to OPTN or UBA3. The KD values presented in (**b**) were calculated via the BIAcore 3000 analysis software (BIAevaluation Version 4.1). $n = 3$ independent experiments. **e** Western blotting analysis of the TLR4, MyD88, (p)-NF-κB, (p)-IRF3, (p)-p38 MAPK levels in Ctrl BMDCs after treatment with SSD (1μM). $n = 3$ independent experiments. **f** Ctrl and *Optn* null BMDCs were treated with SSD, mRNA expression level of *Cd80* and *Cd86* were confirmed by qRT-PCR analysis. $n = 3$ independent experiments, $P_{(\text{Ctrl+SSD-Ctrl})} = 0.0034$, $P_{(Optn^{\Delta DC}\text{-Ctrl+SSD})} = 0.9799$, $P_{(Optn^{\Delta DC}\text{+SSD-Ctrl+SSD})} = 0.9377$ of *Cd80*; $P_{(\text{Ctrl+SSD-Ctrl})} = 0.0009$, $P_{(Optn^{\Delta DC}\text{-Ctrl+SSD})} = 0.9583$, $P_{(Optn^{\Delta DC}\text{+SSD-Ctrl+SSD})} = 0.9998$ of *Cd86*. **g** Ctrl and $Optn^{\Delta CD11c}$ EAE mice (8-week-old, female) were administered daily with SSD (40 mg/kg) or placebo solution intragastrically from the day of immunization. $n = 6$ independent animals for Ctrl+vehicle and Ctrl+SSD groups, $n = 7$ independent animals for $Optn^{\Delta CD11c}$+vehicle and $Optn^{\Delta CD11c}$+SSD groups. Data are presented as means ± SD. **, $P < 0.01$; ***, $P < 0.001$; NS, not significant. One-way ANOVA Tukey's post-hoc analysis. Source data are provided in Source Data file.

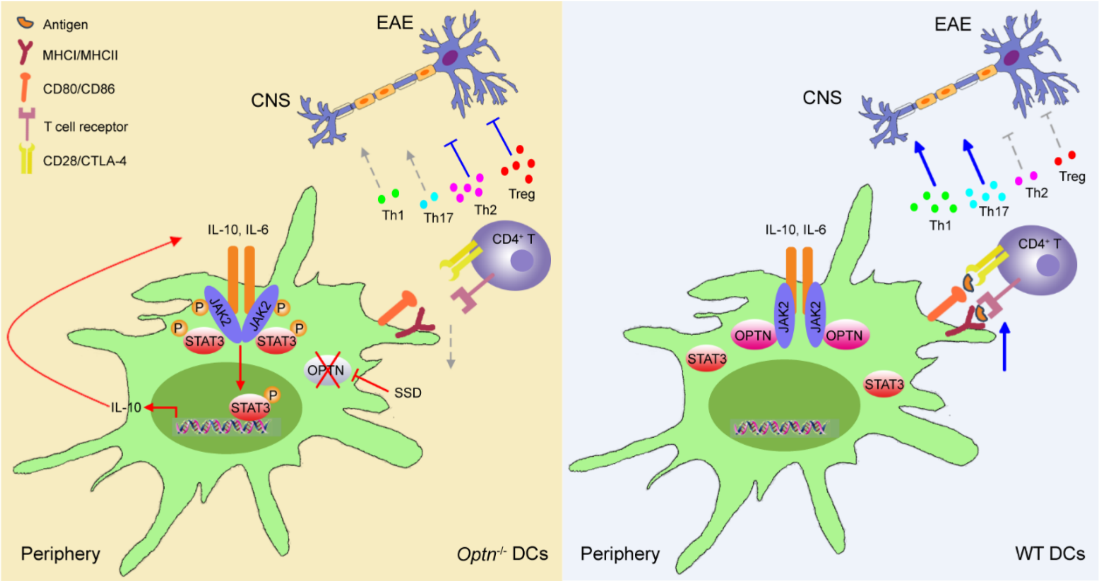

Supplementary Figure 8. Schemes of OPTN function during DC maturation and activation. OPTN promotes the maturation and function of DCs through JAK2-STAT3 signaling pathway, thus driving CD4$^+$ T cells–mediated neuroinflammation in the CNS autoimmune disease.

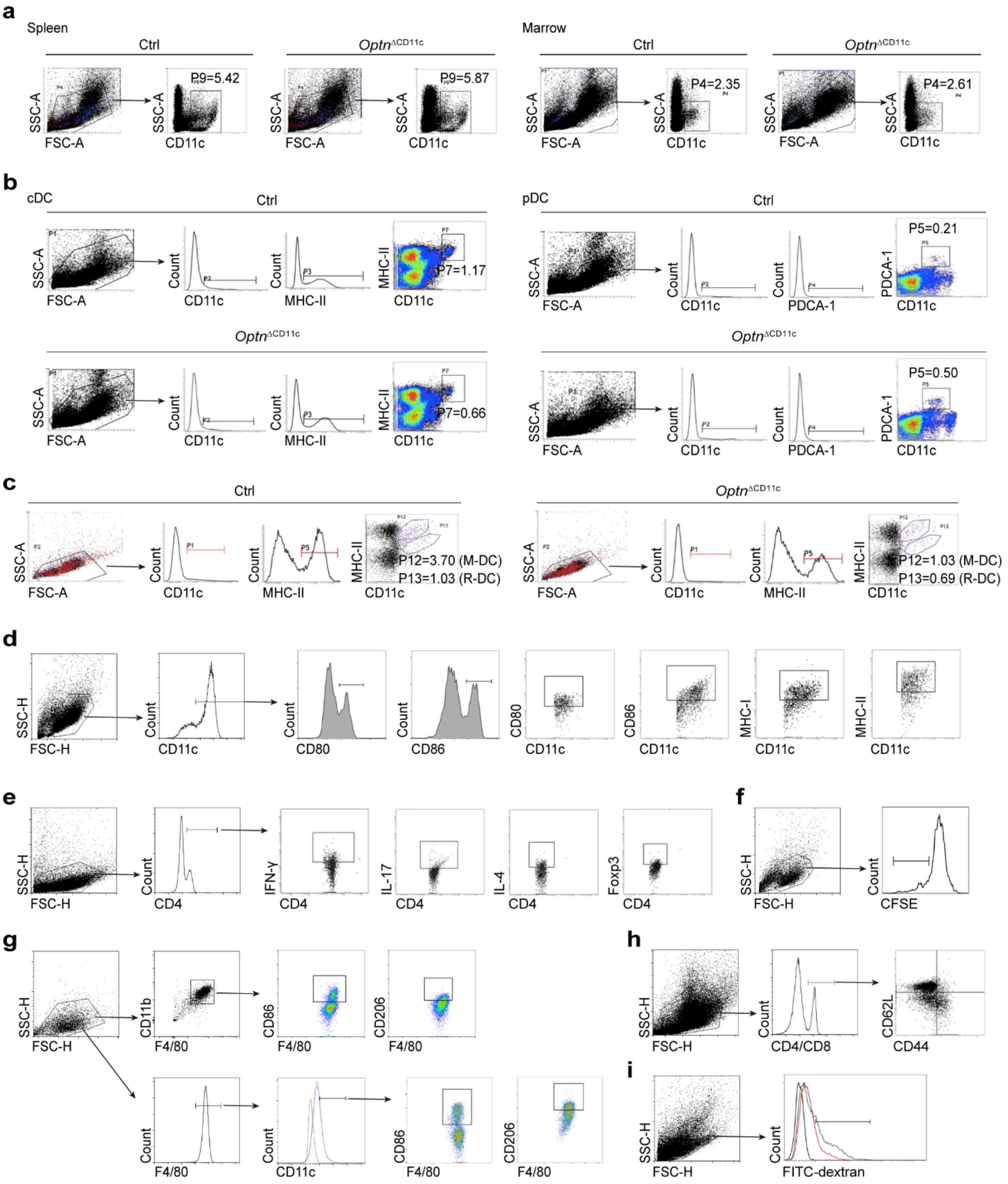

Supplementary Figure 9. Gating strategies used for flow cytometry.
Gating strategies for Fig. 1e (**a**); Fig. 2b (**b**); Fig. 2c (**c**); Fig. 1f-g, 4i, 8c and Supplementary Fig. 4c, 4f (**d**); Fig. 2f, 3e, 3f, 5e-f, 8f, 8j-k and Supplementary Fig. 2f, 2h-i, 5b, 5d, (**e**); Fig. 2e, 8e (**f**); Supplementary Fig. 3a, 3h-i (**g**); Supplementary Fig. 2d-e (**h**); Fig. 2d (**i**).

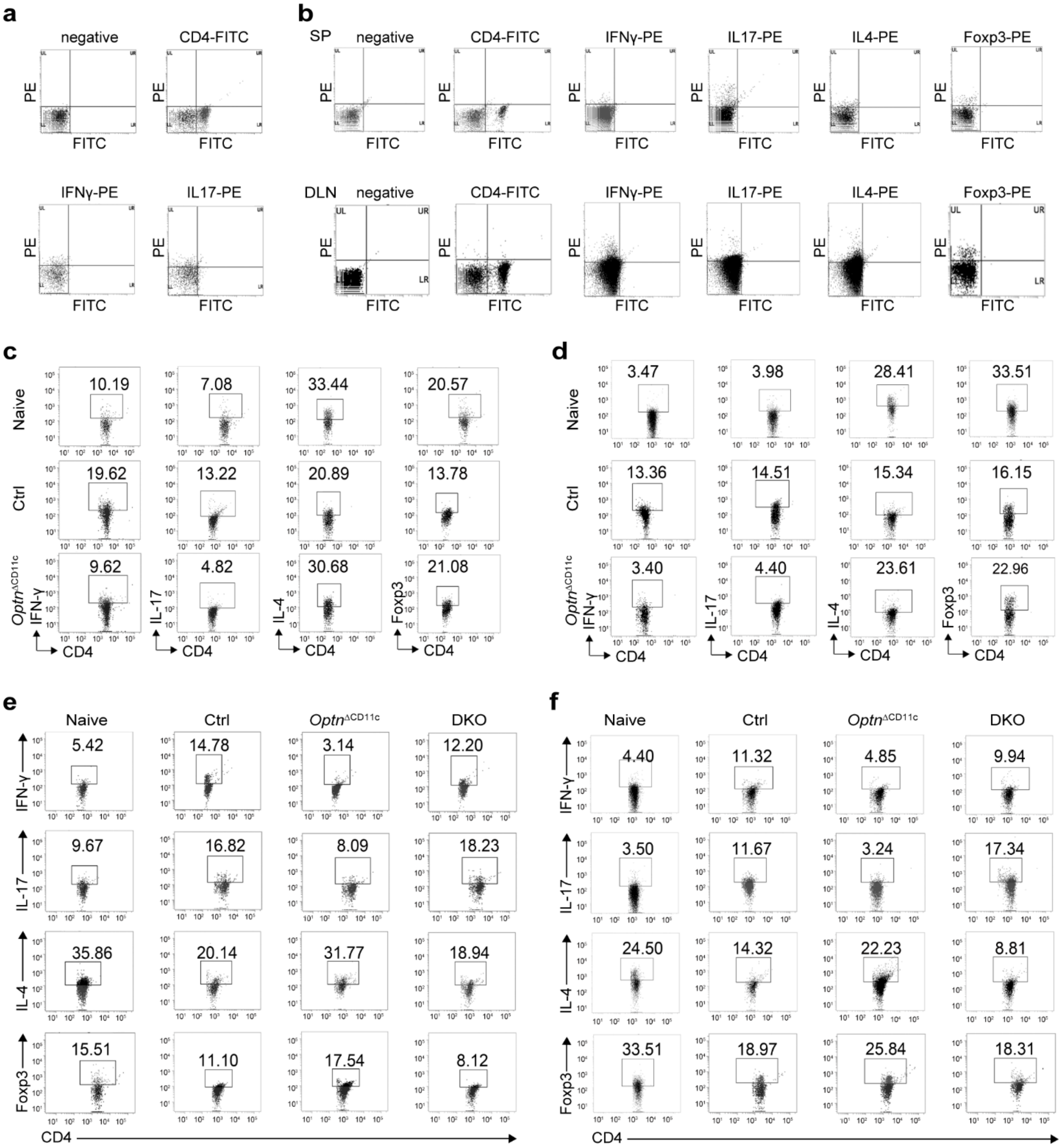

Supplementary Figure 10. Detailed information for flow cytometry analysis of T cells.
a, b Detailed information for analysis of T cells *in vitro* (**a**) and *in vivo* (**b**). **c-f** Analyses of the different CD4$^+$ T subtypes using naïve mice as control group, as for Fig. 3e (**c**), Fig. 3f (**d**), Fig. 5e (**e**), and Fig. 5f (**f**).

Supplementary Tables

Supplementary Table 1. Gene-specific primers used in qRT-PCR experiments.

Gene	Forward primer(5'-3')	Reverse primer(5'-3')
Frs2	TCGGCAGGCAGTGTATATAG	GAAGCGTCTGCTATGCTATGA
Nck1	CCTTCACTCACTGGGAAGTAT	CTCCTTCATGCCCTCTATCAT
Sqstm1	CTCTGGACACGATCCAGTATTC	CTGCTCTACGTGATGCAACTA
Optn	CGCTTAGTGGGCATCTTGTT	CAATCCTGAGCTTGTCGCTATC
Tank	ACAGGCATGCATGGATAGAG	CTGTTGCTCGCGTATTCTATG
Myd88	TCGATGCCTTTATCTGCTACTG	GGTCGGACACACACAACTTA
Stap2	GCTGAGCTGGAAGAGATACTAC	GAGTGAGGATAGAGCTGGAAGA
Caskin1	CCATCCCGCCCATCTATAAT	GAGAGAGGAGAGCAGTCAGTAT
Scimp	CTCCAGATGCTCCTGATGTAT	GTGGTCTGCACTAAGGATACT
Sarm1	TTCCTCCTACCGTCCATCTT	CATCTGCCTCACCTCAGAATAG
Pde4d	ACAGCGACTATGACCTCTCT	TAGCCAAGACCTGAGCATAC
Cd40	GTCACACAGGAGGATGGTATAG	ATAGCAGTTCCAGGGTTCAG
Tnfα	CTACCTTGTTGCCTCCTCTTT	GAGCAGAGGTTCAGTGATGTAG
Il1b	ATGGGCAACCACTTACCTATTT	GTTCTAGAGAGTGCTGCCTAATG
Cxcl9	CAGGCTAGGAGTGGTGAAATG	CAGAGGCCAGAAGAGAGAAATG
Cxcl10	GTGCCAGTCTCACCATCTTTA	GAGGTGTTCTCTTGGTCTTATCC
Vegfa	CACTTCCAGAAACACGACAAAC	TGGAACCGGCATCTTTATCTC
Hif1a	ACCTGGCAATGTCTCCTTTAC	CCAGTGACTCTGGACTTGATTC
Hgf	GGACCATGTGAGGGAGATTATG	ATACCAGGACGATTTGGGATG
Ptgs2	CCTCGTCCAGATGCTATCTTTG	GGCTTCCAGTATTGAGGAGAAC
Socs3	GGTTCTGCTTTGTCTCTCCTATG	TCCCTCAACTCTCTGCCTATT
Il-10	ACAGCCGGGAAGACAATAAC	CAGCTGGTCCTTTGTTTGAAAG
Il-6	CTTCCATCCAGTTGCCTTCT	CTCCGACTTGTGAAGTGGTATAG
Il-13	GCTGAGCAACATCACACAAG	AATCCAGGGCTACACAGAAC
Tgfb2	GGCTATCATATGGCTTGAGATG	CTTCGGGTGAGACCACATATAG
Tgfb3	CGCTACATAGGTGGCAAGAA	CAAGTTGGACTCTCTCCTCAAC
Cd80	AATCCTCCTGCACCAGTATC	CTCTTGGAACCTCAGTGTCTTC
Cd86	TCACCCGATACCTAAGAAGATG	AGAGAGAGGCTGTTGGAGATA
H2-k1	GTGATCTCTGGCTGTGAAGT	GTCTCCACAAGCTCCATGTC
Hl-dra	TCATCCGTCACAGGAGTCAG	TCACAAGAGCTGAGGTGGTG
Arg1	CAGAGGTCCAGAAGAATGGAAG	TCCACCCAAATGACACATAGG
Retnla	GGAGATCCAGAGTGGAGATACT	CCACCTCTTCATTCTTAGGACAG
Mrc1	CAGGTGGCTTATGGGATGTT	CATTTGGGTTCAGGAGTTGTTG
Gapdh	AACAGCAACTCGCACTCTTC	CCTGTTGCTGTAGCCGTATT
