# Supplementary material for: Optineurin modulates the maturation of dendritic cells to regulate autoimmunity through JAK2-STAT3 signaling
Source: Nat Commun. 2021 Oct 27;12:6198. doi: 10.1038/s41467-021-26477-4 (PMC8551263; doi:10.1038/s41467-021-26477-4)
Supplement: Supplementary file 3 — Reporting Summary [file 41467_2021_26477_MOESM3_ESM.pdf]

nature portfolio

Corresponding author(s): Qinjie Weng

Last updated by author(s): Sep 23, 2021

Reporting Summary

Nature Portfolio wishes to improve the reproducibility of the work that we publish. This form provides structure for consistency and transparency in reporting. For further information on Nature Portfolio policies, see our Editorial Policies and the Editorial Policy Checklist.

Statistics

For all statistical analyses, confirm that the following items are present in the figure legend, table legend, main text, or Methods section.

n/a	Confirmed	
☐	☒	The exact sample size (*n*) for each experimental group/condition, given as a discrete number and unit of measurement
☐	☒	A statement on whether measurements were taken from distinct samples or whether the same sample was measured repeatedly
☐	☒	The statistical test(s) used AND whether they are one- or two-sided<br>*Only common tests should be described solely by name; describe more complex techniques in the Methods section.*
☒	☐	A description of all covariates tested
☒	☐	A description of any assumptions or corrections, such as tests of normality and adjustment for multiple comparisons
☐	☒	A full description of the statistical parameters including central tendency (e.g. means) or other basic estimates (e.g. regression coefficient) AND variation (e.g. standard deviation) or associated estimates of uncertainty (e.g. confidence intervals)
☐	☒	For null hypothesis testing, the test statistic (e.g. *F*, *t*, *r*) with confidence intervals, effect sizes, degrees of freedom and *P* value noted<br>*Give P values as exact values whenever suitable.*
☒	☐	For Bayesian analysis, information on the choice of priors and Markov chain Monte Carlo settings
☒	☐	For hierarchical and complex designs, identification of the appropriate level for tests and full reporting of outcomes
☒	☐	Estimates of effect sizes (e.g. Cohen's *d*, Pearson's *r*), indicating how they were calculated

Our web collection on statistics for biologists contains articles on many of the points above.

Software and code

Policy information about availability of computer code

Data collection	Western blot was carried out using enhanced chemiluminescence (ECL) detection reagents (FDbio Science Biotech Co., Ltd., Hangzhou, China). qRT-PCR was carried out using SYBR Green Supermix (Bio-Rad, Hercules, California, USA). Immunofluorescence photography was performed using flurescence microscope (Leica SP8 microscope). Flow cytometry was performed using FACS versus flow cytometry (BD Biosciences). For Fig. 1a analysis, we resorted to the GEO dataset GSE2706, in which human MoDCs were stimulated with LPS for maturation. For Fig. 7j and Supplementary Fig. 6 analysis, we resorted to the dataset GSE27161, which studied STAT3 binding in the genome by ChIP-Seq in DCs.
Data analysis	Flow cytometry analyses were performed by FlowJo (version 10.0.7 win64) and NovoExpress (version 1.5.0). ChIP-seq data were analyzed using Trimmomatic (v0.38), BWA (v0.7.15), MACS (v1.4.1) and IGV (version 2.8.13). Statistical comparisons were performed using GraphPad Prism software (version 8.0.1) and Image J (version 1.8.0).<br>ChIP-seq: We re-analyzed the published STAT3 ChIP-seq data in DCs from GEO dataset (GSE27161). The raw reads were processed through Trimmomatic v0.38 to cut the adapters with the default settings. Then we aligned the high-quality reads to the mm10 reference genome (ftp://ftp.ensembl.org/pub/release-102/fasta/mus_musculus/dna/Mus_musculus.GRCm38.dna.toplevel.fa.gz) using BWA v0.7.15 mem with the default settings. Subsequently, peak calling was performed by MACS v1.4.1 with the following options '-g 1.87e+9 -s 38' and candidate peaks were further filtered with Enrichment ≥ 10-fold and p ≤ 10e-9. Additionally, confident peaks were annotated by ChIP-seeker and Mus musculus gene files (ftp://ftp.ensembl.org/pub/release-102/gff3/mus_musculus/Mus_musculus.GRCm38.102.gff3.gz).

For manuscripts utilizing custom algorithms or software that are central to the research but not yet described in published literature, software must be made available to editors and reviewers. We strongly encourage code deposition in a community repository (e.g. GitHub). See the Nature Portfolio guidelines for submitting code & software for further information.

Data

Policy information about availability of data

All manuscripts must include a data availability statement. This statement should provide the following information, where applicable:

- Accession codes, unique identifiers, or web links for publicly available datasets
- A description of any restrictions on data availability
- For clinical datasets or third party data, please ensure that the statement adheres to our policy

The LPS stimulated human MoDCs array analysis data downloaded and used in Fig. 1a are available in the NCBI Gene Expression Omnibus (GEO) database under accession code GSE2706 [https://www.ncbi.nlm.nih.gov/geo/query/acc.cgi?acc=GSE2706]. The STAT3 genome binding/occupancy profiling of splenic DCs data downloaded and used in Fig. 7j and Supplementary Fig. 6 are available in the NCBI GEO database accession code GSE27161 [https://www.ncbi.nlm.nih.gov/geo/query/acc.cgi?acc=GSE27161]. The data generated in Fig. 1, 2, 3a-b, 3d-g, 4a-h, 4j, 5a-b, 5d-f, 6, 7a-i, 7k, 8a-b, 8d-e, 8g-k and Supplementary Fig. 2a-b, 2d-i, 3, 4b, 4d-e, 4g-h, 5a, 5c, 5e, 7 are provided in the Source Data file. The reporting summary for this Article is available in the Supplementary Information file. All the other data supporting this study are available within this Article, Supplementary Information, Source Data or from the corresponding authors upon reasonable requests.

Field-specific reporting

Please select the one below that is the best fit for your research. If you are not sure, read the appropriate sections before making your selection.

- [x] Life sciences
- [ ] Behavioural & social sciences
- [ ] Ecological, evolutionary & environmental sciences

For a reference copy of the document with all sections, see nature.com/documents/nr-reporting-summary-flat.pdf

Life sciences study design

All studies must disclose on these points even when the disclosure is negative.

Sample size	Sample sizes were indicated in the legend of each Figure and Supplementary Figure. No statistical methods were used to predetermine sample sizes. Sample size choice was based on previous studies in the field (ref: Liu J, Zhang X, Chen K, et al. 2019. DOI: 10.1016/j.immuni.2019.01.021; Zheng J, Sariol A, Meyerholz D, et al. 2020. DOI: 10.1016/j.jaut.2020.102508; Matsumoto M, Baba A, Yokota T, et al. 2014. DOI: 10.1016/j.immuni.2014.10.016; Croxford AL, Lanzinger M, Hartmann FJ, et al. 2015. DOI: 10.1016/j.immuni.2015.08.010; Keller CW, Lünemann JD. 2018. DOI: 10.1080/15548627.2018.1427397; Zhou L, Yao L, Zhang Q, et al. 2020. DOI: 10.1038/s41423-019-0287-0; Yabe R, Chung SH, Murayama MA, et al. 2021. DOI: 10.1038/s41467-020-20307-9).
Data exclusions	No data were excluded.
Replication	The experimental findings were reliably reproduced, for representative data used for statistical analysis, the number of animals or experiments is described in corresponding figure legends.
Randomization	All samples were randomly allocated into experimental groups.
Blinding	For in vivo experiments, the investigators were blinded to group allocation during data collection and analysis.<br><br>For cell-based experiments, immunostaining image analysis, collection of samples were not blinded sometimes due to the cell isolated from the specific animals or the tissue section gained from the specific EAE mice which have obvious differences between groups. The samples were then processed and analysed blindingly in all experiments.

Reporting for specific materials, systems and methods

We require information from authors about some types of materials, experimental systems and methods used in many studies. Here, indicate whether each material, system or method listed is relevant to your study. If you are not sure if a list item applies to your research, read the appropriate section before selecting a response.

Materials & experimental systems

n/a	Involved in the study	
[ ]	[x]	Antibodies
[ ]	[x]	Eukaryotic cell lines
[x]	[ ]	Palaeontology and archaeology
[ ]	[x]	Animals and other organisms
[ ]	[x]	Human research participants
[x]	[ ]	Clinical data
[x]	[ ]	Dual use research of concern

Methods

n/a	Involved in the study	
[x]	[ ]	ChIP-seq
[ ]	[x]	Flow cytometry
[x]	[ ]	MRI-based neuroimaging

Antibodies

Antibodies used

[anti-OPTN] WB, IP, IF, IHC(P), ELISA; Santa Cruz Biotechnology; sc-166576; Mouse monoclonal; L2915
[anti-TLR4] WB, IP, IF, IHC(P); Santa Cruz Biotechnology; sc-293072; Mouse monoclonal; A0220
[anti-Lamin B] Santa Cruz Biotechnology; sc-6216; Goat polyclonal
[anti-JAK2] WB, IP, IHC-P; Cell Signaling Technology; #3230; Rabbit IgG; 11
[anti-p-JAK2(Tyr1007/1008)] WB, IP; Cell Signaling Technology; #3771; Rabbit; 10
[anti-STAT3] WB, IP, IHC-P, IF-IC, F, ChIP; Cell Signaling Technology; #9139; Mouse IgG2a; 10
[anti-p-STAT3(Y705)] WB, IP, IHC-Bond, IHC-P, IF-IC, F, ChIP, ChIP-seq; Cell Signaling Technology; #9145; Rabbit IgG
[anti-MyD88] WB, IP; Cell Signaling Technology; #4283; Rabbit IgG; 4
[anti-p-NF-κB] WB, IP, IF-IC, F; Cell Signaling Technology; #3033; Rabbit IgG
[anti-NF-κB] WB, ICC/IF, IHC-P, IP, FC; Huabio; ET1603-12; Rabbit monoclonal; HM1111
[anti-p38 MAPK] WB, ICC/IF; Huabio; ET1602-26; Rabbit monoclonal; HM0428
[anti-p-p38 MAPK] WB, IHC-P, FC; Huabio; ER1903-01; Rabbit polyclonal; HN0525
[anti-IRF3] WB, ICC/IF, IHC-P, FC; Huabio; ET1612-14; Rabbit monoclonal; HM1125
[anti-p-IRF3(S386)] WB, ICC/IF; Huabio; ET1608-22; Rabbit monoclonal; HN0615
[anti-GAPDH] WB, IHC, ICC/IF, FC, IP; DiagBio Technology; db106; Rabbit polyclonal; P1020
[anti-HA] WB, IP, IHC-P, IF-IC, F, ChIP; Cell Signaling Technology; #3724; Rabbit IgG; 10
[anti-Flag] WB; Cell Signaling Technology; #86861; Rabbit IgG; 2
[anti-Mouse Alexa Fluor 488-coupled secondary antibodies] IF, ICC, IHC; Life Technologies; A-21202; Donkey / IgG
[anti-Human Alexa Fluor 488-coupled secondary antibodies] IF, Flow; Life Technologies; A-11013; Goat / IgG
[anti-Human Alexa Fluor 568-coupled secondary antibodies] IF, ICC, IHC; Life Technologies; A-21090; Goat / IgG

Validation

All primary antibodies used in this study were validated by the manufacture. Validation data / citations can be found on the manufacture website by searching the antibody catalog number provided in materials and methods section of our manuscript.

[anti-OPTN] application: WB, IP, IF, IHC(P), ELISA; species reactivity: mouse, rat, human
https://www.scbt.com/p/optineurin-antibody-c-2?requestFrom=search
[anti-TLR4] application: WB, IP, IF, IHC(P); species reactivity: mouse, rat, human, canine
https://www.scbt.com/p/tlr4-antibody-25?requestFrom=search
[anti-Lamin B] https://www.scbt.com/p/lamin-b-antibody-c-20?requestFrom=search
[anti-JAK2] application: WB, IP, IHC-P; species reactivity: human, mouse, rat
https://www.cellsignal.cn/products/primary-antibodies/jak2-d2e12-xp-rabbit-mab/3230?site-search-type=Products&N=4294956287&Ntt=%233230&fromPage=plp&_requestid=1910914
[anti-p-JAK2(Tyr1007/1008)] application: WB, IP; species reactivity: human, mouse
https://www.cellsignal.cn/products/primary-antibodies/phospho-jak2-tyr1007-1008-antibody/3771?site-search-type=Products&N=4294956287&Ntt=%233771&fromPage=plp&_requestid=1910953
[anti-STAT3] application: WB, IP, IHC-P, IF-IC, F, ChIP; species reactivity: human, mouse, rat, Mk
https://www.cellsignal.cn/products/primary-antibodies/stat3-124h6-mouse-mab/9139?site-search-type=Products&N=4294956287&Ntt=%239139&fromPage=plp&_requestid=1910981
[anti-p-STAT3(Y705)] application: WB, IP, IHC-Bond, IHC-P, IF-IC, F, ChIP, ChIP-seq; species reactivity: human, mouse, rat, Mk
https://www.cellsignal.cn/products/primary-antibodies/phospho-stat3-tyr705-d3a7-xp-rabbit-mab/9145?site-search-type=Products&N=4294956287&Ntt=%239145&fromPage=plp&_requestid=1910998
[anti-MyD88] application: WB, IP; species reactivity: human, mouse, rat, Mk
https://www.cellsignal.cn/products/primary-antibodies/myd88-d80f5-rabbit-mab/4283?site-search-type=Products&N=4294956287&Ntt=%234283&fromPage=plp&_requestid=1911023
[anti-p-NF-κB] application: WB, IP, IF-IC, F; species reactivity: human, mouse, rat, Hm, Mk, Pg
https://www.cellsignal.cn/products/primary-antibodies/phospho-nf-kb-p65-ser536-93h1-rabbit-mab/3033?site-search-type=Products&N=4294956287&Ntt=%233033&fromPage=plp&_requestid=1911076
[anti-NF-κB] application: WB, ICC/IF, IHC-P, IP, FC; species reactivity: human, mouse
http://www.huabio.cn/product/NF-kB-p65-antibody-ET1603-12
[anti-p38 MAPK] application: WB, ICC/IF; species reactivity: human, mouse, rat
http://www.huabio.cn/product/p38-antibody-ET1602-26
[anti-p-p38 MAPK] application: WB, IHC-P, FC; species reactivity: human, mouse, rat
http://www.huabio.cn/product/Phospho-P38-MAPK-Thr180-Tyr182-antibody-ER1903-01
[anti-IRF3] application: WB, ICC/IF, IHC-P, FC; species reactivity: human, mouse, rat
http://www.huabio.cn/product/IRF3-antibody-ET1612-14
[anti-p-IRF3(S386)] application: WB, ICC/IF; species reactivity: human
http://www.huabio.cn/product/Phospho-IRF3-S386-antibody-ET1608-22
[anti-GAPDH] application: WB,IHC,ICC/IF,FC,IP; species reactivity: human, mouse, rat
http://www.diagbio.com/prodetail.aspx?caid3=73&pid=31405
[anti-HA] application: WB, IP, IHC-P, IF-IC, F, ChIP; species reactivity: ALL
https://www.cellsignal.cn/products/primary-antibodies/ha-tag-c29f4-rabbit-mab/3724?site-search-type=Products&N=4294956287&Ntt=ha-tag+%28c29f4%29+rabbit+mab&fromPage=plp
[anti-Flag] application: WB; species reactivity: ALL
https://www.cellsignal.cn/products/antibody-conjugates/dykddddk-tag-d6w5b-rabbit-mab-binds-to-same-epitope-as-sigma-s-anti-flag-m2-antibody-hrp-conjugate/86861?site-search-type=Products&N=4294956287&Ntt=%2386861&fromPage=plp&_requestid=510

Eukaryotic cell lines

Policy information about cell lines

Cell line source(s)	BMDCs and BMDMs were prepared from the bone marrow of 6~8-week-old C57BL/6 mice; CD11c+ DCs and CD4+ T cells were isolated from the spleen and lymph nodes of 6~8-week-old C57BL/6 mice; human monocyte-derived dendritic cells and peripheral blood mononuclear cells were isolated from human blood samples; HEK293 cells were purchased from Invitrogene.
Authentication	DCs were authenticate by Flow cytometry analysis for CD11c staining. BMDMs were authenticate by Flow cytometry analysis for F4/80 and CD11b staining. CD4+ T cells were authenticate by Flow cytometry analysis for CD4 staining. Other cells were used without modification once received from supplier and therefore were not authenticated.
Mycoplasma contamination	All cell lines tested negative for mycoplasma contamination.
Commonly misidentified lines (See ICLAC register)	No commonly misidentified lines were used.

Animals and other organisms

Policy information about studies involving animals; ARRIVE guidelines recommended for reporting animal research

Laboratory animals	As reported in Methods section of “Mice” for the information of animal species and strains. The mouse strains used in this study were generated and maintained on a mixed C57Bl/6 background.<br>C57BL/6 mice were obtained from Beijing Vital River Laboratory Animal Technology Co., Ltd.<br>CD11c-Cre mice were acquired from The Jackson Laboratory.<br>Optnfl/fl mice were gifted by Pro. Ronggui Hu.<br>Stat3fl/fl mice were obtained from the Shanghai Model Organisms Center.<br>OT-II mice were given by Pro. Lie Wang as gift.<br>All mice were housed in specific pathogen-free environment at 21 ± 1 °C and 60 ± 5% humidity, with a 12-h light/dark cycle. Experimental and control animals were bred separately. All mice were used at 6-8 weeks of age. Female mice were used for EAE model, female or male mice were used for in vitro experiment.
Wild animals	Wild animals were not involved in this study.
Field-collected samples	Field-collected samples were not involved in this study.
Ethics oversight	All animal use and studies were performed in compliance with all relevant ethical regulations, and were approved by the Institutional Animal Care and Use Committee (IACUC) at Zhejiang University.

Note that full information on the approval of the study protocol must also be provided in the manuscript.

Human research participants

Policy information about studies involving human research participants

Population characteristics	Healthy donor are 10~30 years old males and females.
Recruitment	Blank human peripheral blood was collected from the Second Affiliated Hospital of School of Medicine, Zhejiang University. Samples from healthy people who showed no history of major diseases and had normal biochemical indicators and blood routine after physical examination were involved in this cohort. Ailing peripheral blood was excluded in the study to avoid the influence of the diseases to their peripheral blood or MoDCs.
Ethics oversight	Volunteers provided informed consent for this study. All experiments were approved by the Human Subject Research Ethics Committee of the Second Affiliated Hospital of School of Medicine, Zhejiang University (No. Yan2016-003).

Note that full information on the approval of the study protocol must also be provided in the manuscript.

Flow Cytometry

Plots

Confirm that:

- [x] The axis labels state the marker and fluorochrome used (e.g. CD4-FITC).
- [x] The axis scales are clearly visible. Include numbers along axes only for bottom left plot of group (a 'group' is an analysis of identical markers).
- [x] All plots are contour plots with outliers or pseudocolor plots.
- [x] A numerical value for number of cells or percentage (with statistics) is provided.

Methodology

Sample preparation	The phenotypes of DCs and BMDMs were determined by flow cytometry. For cell surface staining, single-cell suspensions were incubated for 15 min at 4 °C with PE-anti-CD11c (117308, 0.2 μg/mL), FITC-anti-CD80 (104706, 0.2 μg/mL), PE/Cy7-anti-CD86 (105014, 0.2 μg/mL), FITC-anti-MHC-I (114606, 0.2 μg/mL), PerCP/Cy5.5-anti-MHC-II (107626, 0.2 μg/mL), FITC-anti-F4/80 (123108, 0.2 μg/mL), PerCP/Cy5.5-anti-CD11b (101228, 0.3 μg/mL), PE-anti-CD206 (141706, 0.2 μg/mL), APC-anti-CD115 (135509, 0.4 μg/mL), FITC-anti-PDCA-1 (127007, 0.3 μg/mL), PerCP/Cy5.5-anti-CD44 (103032, 0.2 μg/mL), PE-anti-CD62L (104407, 0.2 μg/mL) (all purchased from Biolegend). Samples were washed and then analyzed by FACS versus flow cytometry (BD Biosciences).<br>For intracellular staining of cytokines, single-cell suspensions from DLN and spleen of the indicated EAE mice were stained with FITC-anti-CD4 (553047, BD Biosciences, 0.2 μg/mL) first, followed by staining with PE-anti-IFN-γ (554412, BD Biosciences, 0.3 μg/mL), PE-anti-IL17A (506904, Biolegend, 0.3 μg/mL) and PE-anti-IL4 (12-7041-82, eBioScience, 0.4 μg/mL) using Cytofix/Cytoperm kit (BD Biosciences) according to the manufacturer's protocol. Intranuclear staining with PE-anti-Foxp3 (126404, Biolegend, 0.4 μg/mL) was performed using a Fixation/Permeabilization kit (eBioscience) according to the manufacturer's protocol.
Instrument	BD FACSVERS and ACEA NovoCyteTM
Software	FlowJo (10.0.7 win64), NovoExpress (1.5.0)
Cell population abundance	By stained with specific fluoresence-labeled antibodies, cells were separated into different parts and cytometry can calculate the cell fractions.
Gating strategy	For DCs detection, CD11c-PE was used to label DCs, and CD80-FITC, CD86-PE/Cy7, MHC-I-FITC and MHC-II-PerCP/Cy5.5 were used to label mature DCs. PBS-treated cells were stained with/without single antibodies to determine gate.<br>For BMDMs detection, F4/80-FITC and CD11b-PerCP/Cy5.5 were used to label BMDMs, and CD86-PE/Cy7 or CD206-PE was used to label M1 or M2 BMDMs. PBS-treated cells were stained with/without single antibodies to determine gate.<br>For CD4+ T detection, CD4-FITC was used to label CD4+ T cells, and IFN-γ-PE, IL17A-PE, IL4-PE and Foxp3-PE were used to label Th1, Th17, Th2 and Treg cells. PBS-treated cells were stained with/without single antibodies to determine gate.<br>All gating strategies were showed in Supplementary Figure 9.

☒ Tick this box to confirm that a figure exemplifying the gating strategy is provided in the Supplementary Information.
